# Competition in the Dutch hospital sector: an analysis of health care volume and cost

**DOI:** 10.1007/s10198-016-0762-9

**Published:** 2016-02-01

**Authors:** Y. J. F. M. Krabbe-Alkemade, T. L. C. M. Groot, M. Lindeboom

**Affiliations:** 1 Department of Accounting, Faculty of Economics and Business Administration, VU University Amsterdam, De Boelelaan 1105, 1081 HV Amsterdam, The Netherlands; 2 Department of Economics, Faculty of Economics and Business Administration, VU University Amsterdam, De Boelelaan 1105, 1081 HV Amsterdam, The Netherlands

**Keywords:** Prospective payment system, Hospital competition, Hospital costs, Hospital production, I11, I18, C23

## Abstract

This paper evaluates the impact of market competition on health care volume and cost. At the start of 2005, the financing system of Dutch hospitals started to be gradually changed from a closed-end budgeting system to a non-regulated price competitive prospective reimbursement system. The gradual implementation of price competition is a ‘natural experiment’ that provides a unique opportunity to analyze the effects of market competition on hospital behavior. We have access to a unique database, which contains hospital discharge data of diagnosis treatment combinations (DBCs) of individual patients, including detailed care activities. Difference-in-difference estimates show that the implementation of market-based competition leads to relatively lower total costs, production volume and number of activities overall. Difference-in-difference estimates on treatment level show that the average costs for outpatient DBCs decreased due to a decrease in the number of activities per DBC. The introduction of market competition led to an increase of average costs of inpatient DBCs. Since both volume and number of activities have not changed significantly, we conclude that the cost increase is likely the result of more expensive activities. A possible explanation for our finding is that hospitals look for possible efficiency improvements in predominantly outpatient care products that are relatively straightforward, using easily analyzable technologies. The effects of competition on average cost and the relative shares of inpatient and outpatient treatments on specialty level are significant but contrary for cardiology and orthopedics, suggesting that specialties react differently to competitive incentives.

## Introduction

Total health care expenditures in the Netherlands increased from €6.5 billion in 1972 to €89.7 billion in 2011 [1]. The dramatic growth in Dutch health care costs is similar to health care cost increases experienced in other countries. Before 1983, health care providers were retrospectively reimbursed by a fee-for-service system. This system relied on a fee schedule of hospital services with prices regulated by the National Tariff Agency (NTA). Because hospital production was not regulated in this system, the volume and the health care expenditures increased as a result. The Dutch government tried to control the increasing expenditures by implementing several budgeting systems between 1983 and 1988, ranging from a budget system based on previous year expenditures, to a function-based budget system existing of a combination of (semi-) fixed and variable budget parameters. In 1995, a budget system for physicians was introduced to bring the incentives of both reimbursement systems in line with each other. There were some drawbacks to these systems. First, there was no direct relation between tariffs of the budget parameters and the actual costs of realized care activities. Second, both systems ended up with waiting lists. These outcomes led to broad support for incentive-based reforms and the introduction of managed competition in 2005 [2].

Managed competition is a system in which care consumers can freely choose among health insurers, health insurers contract or integrate with health care providers, and governments regulate competition within both health insurer and health care provider markets to ensure the public goals of universal access to affordable, quality care [3–5]. Market competition in the Dutch health care market implies that insurers and hospitals are allowed to negotiate freely about health care volume, price and quality, and to contract selectively. A competitive prospective payment system called the Diagnosis Treatment Combination system was introduced to incentivize hospitals to control their costs and improve quality and transparency.

Market competition has been implemented incrementally: In 2005 10 % of the health care products, mostly the more standardized treatments, were transferred from the budgeting system into the market system. This percentage was expanded to 20 % in 2008, to 34 % in 2009 and eventually to 70 % in 2012. In the new system, new providers are allowed to enter the hospital market, which resulted in a strong growth of Independent Treatment Centers (ITCs) providing high-volume elective care.

This paper analyzes the impact of market competition on health care volume and costs. The implementation of the system is a ‘natural experiment’ and its incremental introduction offers the opportunity to compare the performance of experimental product groups in the competitive market with the performance of control groups that are still in the budgeting system.

Our contribution to the literature is twofold. First, we tested the effects of market competition in a unique setting in which all hospitals of the Netherlands are included. Within this setting, over the years we observed the implementation of a competitive prospective payment system for some care products, while others remained in the pre-reform budget financing system. This allows us to use the difference-in-difference method to assess the effects on volume and costs. Secondly, our unique database allowed us to measure care intensity more completely and reliably, where previous studies had to rely on crude proxies, e.g. number of admissions or length of stay. Our database contains hospital discharge data of more than 800,000 Diagnosis Treatment Combinations (in Dutch: DBCs) of individual patients, including detailed care activities provided by medical specialists and support staff. This system was systematically applied by all Dutch hospitals in the selected period of 2006–2008.

This paper is organized as follows. The following section describes the microeconomic considerations. The section “Problem formulation” discusses the Dutch policy context and the hypotheses. The section “Data and model specification” explains the difference-in-difference models used, the variables and the data. The section “Results” presents the results of the research. The conclusion is provided in the section “Conclusion”. The last section “Discussion and limitations” describes the results and address several limitations.

### Microeconomic considerations

Health care markets deviate from perfectly competitive markets. The industrial organization of health care views hospitals as entities operating in an environment of monopolistic competition. Each hospital sells a differentiated product, while a patient’s preference for a health care provider is determined on real or perceived differences in ability and the idiosyncratic match. This limits substitutability of health care providers. Consequently, health care providers may increase price or decrease some quality attributes without losing all their patients to other providers [6]. In other words, product prices are not perfectly elastic, leading to unequal provider power over price and production. Furthermore, the health care market suffers from asymmetric information problems at different levels, e.g., between patient and physician and between hospital and insurer. Information asymmetry problems may lead to over- or under-consumption of health care. These problems may be aggravated by adverse selection and moral hazard problems in both the hospital market and the health insurance market [6–9]. We expect health care demand, either expressed by privately insured patients, their referring physicians, or as intermediated by managed care, to be negatively related to price. The price elasticity of demand in specific health care markets is considered to be dependent on hospital characteristics, quality of the health product, and both supply and demand conditions.

When a government administratively determines prices, the only option left to hospitals is to compete on quality in order to attract patients. Studies from the UK show that with fixed prices and more competition, quality increases [10–12]. However, setting the appropriate price is crucial, as too-high prices may motivate hospitals to increase costs by providing additional unnecessary medical services and amenities. In the situation where both prices and quality vary, the market outcome will depend on the relative size of the elasticity of demand with respect to quality and price. Gaynor and Town [13] show the relationship between quality and price with the ‘Dorfman Steiner condition’:

$$z = \frac{p}{d}\frac{{\varepsilon_{z} }}{{\varepsilon_{p} }},$$

where *z* is quality, *p* price, *d* the marginal cost of quality, *ε*_*z*_ the quality elasticity of demand and *ε*_*p*_ the price elasticity of demand. This formula shows that if, in a market with variable prices, the quality elasticity of demand increases or price elasticity of demand decreases, the quality will increase. On the other hand, quality will decrease when quality elasticity of demand decreases or price elasticity of demand increases [13].

The above holds true when health insurers are perfectly informed about price and quality. However, in most health care markets and also in our observation period, quality information is relatively limited [14]. When quality is not perfectly observable, health consumers will not react to quality differences. Thus, the absolute quality elasticity of demand is low. When price information becomes less noisy because of the introduction of health care production systems, like Diagnosis Related Group (DRG) or DBC systems, and when price differences between care suppliers become transparent, health care demand will be more price-elastic. This will drive health care prices and price–cost margins down [15].

With lower prices, hospitals can only maintain price–cost margins by reducing costs. Hospitals can reduce costs in several ways. First, hospitals can obtain cost cuts by performing fewer activities per treatment, by performing less expensive activities for a given diagnosis or by changing the treatment choices for a given diagnosis. Concerning the latter, for instance, specialists could shift from inpatient treatments to daycare or outpatient treatments. Ultimately this depends on cost-price margins of different treatments. Since we do not observe these, it will be difficult to predict a priori just how treatment choices will change.

When prices are set by the government in a budgeting system, hospital production is maximized to the budgeted volumes, since production beyond the budget will not be reimbursed. When the budgeting system is replaced by a market system in which hospitals are reimbursed via payment-for-performance, there is no upper limit to health production. Hospitals will forecast the demand of care and determine the required input capacity. However, when the actual demand exceeds the predicted demand, the input capacity will be too low. Hospitals have to decrease their resources, which could lead to poor quality. When the actual demand is beyond the predicted demand, the input capacitance will be too large which may lead to inefficiency or congestion [16].

### Problem formulation

The impact of competition in the health care sector depends on the purchasing strategy of health insurers, the way hospitals compete with each other, the rules under which competition takes place and design of the reimbursement systems of hospitals and physicians. Hospital competition can be divided into patient-driven competition and payer-driven competition. In patient-driven competitive systems, the patient or his physician chooses a hospital for treatment. When patients are insured, their demands are not price-sensitive because insured patients do not incur high out-of-pocket expenses [6, 17]. Under patient-driven competition, hospitals are generally reimbursed on a fee-for-service basis. The empirical literature is inconclusive about the impact of patient-driven competition on prices, costs and volume of care. Early US studies show that more competition among hospitals leads to lower prices and costs [17–19]. The negative association of market concentration with prices appears to be mediated by the level of price sensitivity of demand [20]. Other studies conclude that patient-driven competition with fee-for-service reimbursement leads to increasing prices and expenses, because hospitals compete on quality services and amenities, driving health care costs up due to the provision of duplicated capital-intensive services [21–25]. This form of competition, which is also known as the theory of the ‘medical arms race’ (MAR), drives up prices and health care costs. When the number of hospitals in a health care market increases, each patient or physician gets more bargaining power and can play the hospitals off against each other. In this way, health care consumers can extract more services and a higher quality of care from the providers [6, 26, 27].

Payer-driven competition is a system in which the insurer, not the patient or his physician, selects the health care provider and decides about care consumption. In a payer-driven competitive market, purchasers may restrict patient’s hospital choice by selectively contracting hospitals based on price benefits, quality requirements and service levels rather than idiosyncratic advantages. High purchaser concentration leads to monopsony power, which generally results in lower price–cost margins for hospitals, especially in competitive health care markets [17]. Propper et al. reviewed all price studies about payer-driven competition in the UK and concluded that there are large price differences between health care providers offering comparable care. Studies that examine the relationship between hospital competition and prices show that more competition results in lower prices for low-cost and elective care medical specialties [15]. Söderlund analyzed the relationship between competition and average costs per inpatient episode for acute care hospitals and found a positive, but non-significant relationship between market concentration and costs [28].

The Dutch health care market is a combination of patient- and payer-driven competitive systems. Almost all patients in the Netherlands are insured because of the mandatory health insurance system, which includes virtually no co-payments and a low optional deductible [29]. Health insurers ‘manage competition’ by negotiating with health care providers about price, volume and quality of care for their enrollees. Most insurance companies have contracted almost every hospital and therefore selective contracting is hardly ever used. Patients may freely select the hospital and they are relatively price insensitive because of the insurance policy they hold.

To understand the incentives within the reformed Dutch hospital market, insight is needed into the hospital payment systems before and after the implementation of the Dutch health care reforms in 2005, because during our research period the old system still existed for a part of the hospital production. Before the health care reforms, hospitals and physicians were reimbursed by separate payment systems. Hospitals received a budget, which consisted of a combination of (semi-) fixed and variable budget parameters. The tariffs of these parameters were fixed. Only the volume of variable budget parameters was determined by negotiations between hospital and health insurer. Variable budget parameters are, for example, outpatient visits, day care, and inpatient days. When a hospital exceeded or underspent the budget, an adjustment rate balanced the hospital’s budget retrospectively. Physicians also received a budget for their services, the so-called lump sum system. Both systems stimulated hospitals and physicians to control hospital expenditures. Hospitals and physicians had no incentive to treat more patients or to increase the number of activities per patient. The advantage of the budget system is that it led to expenditure control. The disadvantage is that waiting lists were being created and waiting times were getting longer, which had negative consequences for patient needs and quality of care [2].

The incentives of the Diagnosis Treatment Combinations (DBC) system should motivate hospitals and physicians to treat all patients that need hospital care and to provide only necessary services. This Diagnosis Related Group (DRG)-based system has the incentive to increase the number of cases and decrease the number of services per case. Hospitals negotiate about the volume and price and quality of 10 % of the hospital expenses, the so-called B-segment. However, relevant information was scant. We therefore expect that health insurers will compete more on price, which will lead to lower costs or number of activities per case. However, we must not forget that price competition could also result in lower quality [30], but we could not observe quality differences during our research period. Because physicians were paid per DBC and health insurers were not able to select contracting hospitals, we expect volume increase in the B-segment. For 90 %, the A-segment, the FB budget and lump sum budget still existed. The A-segment still has the incentives to control the number of cases and hospital services in order to not exceed the budget. The B-segment gradually increased to 20 % of the hospital expenses in 2008, 34 % in 2009 and to 70 % in 2012.

In this study, we focus on the short-term effects of market competition. Based on the above-mentioned considerations, our hypotheses are as follows:

#### **Hypothesis 1**

The introduction of market competition in the Dutch quasi-market system (the B segment) will lead to higher production volume of DBCs.

#### **Hypothesis 2**

The introduction of market competition in the Dutch quasi-market system (the B segment) will lead to lower average costs or to fewer health care activities per DBC than under the budgeting system.

### Data and model specification

**Fig. 1:**
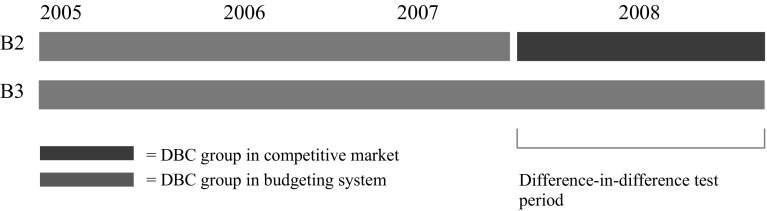
Graphic representation of treatment group (B2) and control group (B3)

The treatment group B2 in our difference-in-difference model contains DBCs that were transferred from the budget-based system into the market competition system in 2008. The control group B3 consists of products that remained in the budget-based system but entered the competitive system in the next round in 2009. A graphic representation of the two groups is depicted in Fig. 1.

#### Assignment of treatment and control group

The introduction of market coordination in the Netherlands followed an incremental process, in which four groups of DBCs were transferred from the budgeting system into the market system: the first started in 2005, the second group followed in 2008, the third in 2009 and the last group in 2012.

Mainly low-complexity care DBCs with clear product definitions and transparent information about quality and price were eligible to be transferred to the market system. The Dutch health care market for the selected low-complex DBCs also needed to be sufficiently efficient, which meant a market in which many suppliers are active, in which patients can freely select their preferred health care provider and which offers ample opportunities for new providers to enter the market. The markets for the selected DBCs also needed to have low market failures such as negative external effects and high transaction costs [31]. In this market hospitals provide services to all patients that need hospital care for the lowest cost and an accepted quality level. Hospitals are only trying to minimize the number of services per case without jeopardizing standard quality and are not able to manipulate the number of cases [32].

The implementation process started with the low-complexity care DBCs and the level of complexity gradually rose as the implementation process evolved. For our difference-in-difference analysis we selected the second DBC group as the experimental group and the third DBC group as the control group. We did not use the first DBC group in order to avoid the irregularities related to the first implementation and the non-existence of comparable production data in the years prior to 2005. We expected that providers did have some market experience by the time the second DBC group was transferred, which may have led to more effective market responses. We also expected the second and third groups to be comparable in level of health care complexity and relevant market characteristics, which makes them suitable for our difference-in-difference analyses.

#### DIS data 2006–2008

The production and cost data are taken from the DBC Information System (DIS) for the years 2006 and 2008. In the analyses, we use 2006 as a pre-reform year and 2008 as a post-reform year. However, hospitals may have anticipated the change in 2007, which was the reason for using 2006 as the pre-reform year. The DIS national database consists of DBC data and patient-level care activities from all Dutch hospitals. University hospitals and specialty hospitals are excluded from the database. Six general hospitals are also excluded because three denied permission to use their data and three appeared to have incomplete data in the DIS. The B2 and B3 segments consist of 3327 and 2123 unique DBC codes, respectively. We selected high-volume diagnoses and excluded DBCs with ‘follow-up treatment’ type of care. This resulted in 11 diagnoses: 46 DBCs of 4 medical specialties (cardiology, dermatology, gastroenterology and orthopedics) in the B2-segment, and 10 diagnoses with 54 DBC codes of 3 specialties (cardiology, dermatology and orthopedics) in the B3 segment. Our dataset contains 974,592 DBC records, representing 25 % of the total cases of the B2 and B3 segments. The selected DBC codes are depicted in Table 1.

**Table 1:** Sample selected DBCs of treatment group (B2) and control group (B3)

Segment	Specialty	Diagnosis code	Diagnosis name	Treatment codes
B2	Cardiology	302	Chronic heart failure	111,112, 113
B2	Cardiology	401	Cardiac atrium fibrillation	111, 112, 113
B2	Cardiology	402	Other cardiac arrhythmia originated in cardiac atrium	111, 112, 113
B2	Cardiology	404	Cardiac impulse disorder	111, 112, 113
B2	Cardiology	409	Other cardiac arrhythmia	111, 112, 113
B2	Dermatology	20	Psoriasiform dermatoses	11, 81, 82, 92, 93
B2	Gastroenterology	601	Inflammatory bowel disease	101, 102, 103, 202, 203
B2	Gastroenterology	602	Ulcerative colitis	101, 102, 103, 202, 203
B2	Orthopedics	1630	Carpal tunnel syndrome	211, 212, 213, 216
B2	Orthopedics	1805	Meniscal injury	211, 212, 213, 216, 223, 226
B2	Orthopedics	1820	Anterior cruciate ligament injury	211, 212, 213, 216, 223, 226
B3	Cardiology	202	Angina pectoris, stable	101, 102, 103
B3	Cardiology	203	Angina pectoris, unstable	101, 102, 103
B3	Cardiology	302	Chronic heart failure	101, 102, 103
B3	Cardiology	401	Cardiac atrium fibrillation	101, 102, 103
B3	Cardiology	402	Other cardiac arrhythmia originated in cardiac atrium	101, 102, 103
B3	Dermatology	14	Malignant dermatosis	11, 21, 31, 41, 51, 81, 82, 92, 93
B3	Dermatology	22	Ulcus cruris	11, 31, 71, 92, 93
B3	Orthopedics	1240	Cervical stenosis with myelopathy	111. 112, 113, 211, 212, 213, 216, 223, 226
B3	Orthopedics	1350	Spinal stenosis	111, 112, 113, 211, 212, 213, 216
B3	Orthopedics	1803	Loosening/infection malposition knee arthroplasty	111, 112, 113, 211, 212, 213, 216, 223, 226

We tested the dataset for the completeness, consistency and reliability of the data. To check the completeness, we removed DBC cases when the DBC information was incomplete (missing parts of DBC codes, episode numbers and dates), erroneous (non-matching episode start date, activity dates and end date; or activity codes containing zeros or negative values), or empty (not containing any care activity information). To screen the consistency, different synonymous treatment code names were pooled into the same categories and some “exotic” DBCs like “urgent care” DBCs that only existed for a short period of time were excluded from the database. To check the reliability of the data, we excluded extreme high cost outliers from the database. The procedures followed led to the removal of 16.5 % of the cases. Our final dataset contains production data of 72 Dutch general hospitals, representing 75 % of all Dutch general hospitals. The final dataset contains 814,192 DBCs, from which 390,770 DBCs are from 2006 and 413,422 DBCs are from 2008.

#### Dependent variables

We use four outcome measures. The DBC volume is the number of registered and charged cases for each DBC care product per hospital (“DBC volume”). DBC activities are the average number of care activities in a DBC per hospital (“DBC activities”). This variable is calculated as the total number of activities produced in a hospital for a DBC care product divided by the total number of DBC cases delivered. The total cost of DBCs produced (“DBC total cost”) is calculated by multiplying the average cost per DBC by the number of DBC cases produced for each hospital. The DBC average cost (“DBC average cost”) is the average cost per DBC delivered per hospital. The DBC average cost is determined by the sum of the costs of all activities represented in the DBC *care profile.* The care profile contains a number of activities such as (outpatient) visits, admissions, bed days and type and volume of care activities. The cost of each activity is calculated by multiplying the actual number of care activities delivered in a DBC by a health care provider with standardized national prices from 2005. The national activity prices are derived from cost prices of 39 frontrunner hospitals that participated in the development of the DBC system [33]. The DBC can be linked to the care profile using unique identification numbers. After linking the full cost prices to the activities, all costs of product activities are aggregated to the total costs per DBC. The average DBC cost in each hospital is then calculated in order to arrive at cost differences for each DBC between hospitals. Price levels in 2005 are also applied to the 2008 DBCs. This is done in order to avoid the impact of price differences on cost information, so that cost differences are only caused by differences in volume and composition of care activities. Because we used a refined proxy for cost based on patient level activities, we were able to analyze changes in resource consumption. This means that we could examine changes in the number of inputs or combination of inputs. However, these costs might differ from the actual hospital cost. The DBC average cost, therefore, does not represent the actual costs incurred by hospitals, but represents care intensity whereby each care activity is weighted according to its relative cost. In this way, substitution between activities with different cost prices can be detected, and differences in resource use become visible. However, because actual hospital costs are not available we cannot measure forms of X-inefficiency as a result of the reform.

To correct for right-skewed distributions we used the natural logarithm of the variables’ average costs, volume and average number of activities [34].

#### Independent variables

##### Treatment variables

A market competition dummy is used to indicate whether a DBC_*i*_ is in the B2 group, which will at some point of time enter the market competition system (value 1), or in the B3 group, which will remain in the budget system (value 0). The model also includes a year dummy for 2008. The treatment variable is an interaction variable of the market competition and year dummies to identify the B2 DBCs that went into the competitive market in 2008.

##### Environmental and hospital characteristics

Hospitals could react differently to competition because of their environmental factors such as market structure, insurer concentration, ageing population or hospital characteristics such as type and size of hospital [32]. To measure market concentration of hospitals, we calculate each hospital-DBC combination using the Herfindahl–Hirschman index (HHI), which is the sum of squared market shares of the B2 and B3 volume of the hospitals competing in the same market. We define the relevant market of a hospital based on a patient’s maximum travel time of 15 min to a hospital using ZIP code-4 information, which means that the maximum travel time between two hospitals is 30 min. The insurance market concentration variable is determined by the HHI of the market shares of the insurance companies for each hospital and each DBC. Health insurers are grouped into 11 major (holding) companies. We have grouped ten small health insurance companies together in a category “other”, which together occupy 0.62 % of the market share. The data on health insurers was derived from the DIS database. For each hospital, we identified the number of DBCs invoiced to each of the health insurers. Hospitals with more than 10 % of DBCs that could not be invoiced to a health insurer are excluded. A low hospital insurer concentration index means that the total hospital production regarding a certain DBC is equally financed by a large number of insurance companies. A high hospital-insurer concentration indicates monopsonic power held by a few insurance companies financing a large portion of care regarding a given DBC. We furthermore include a dummy for type of hospital to control for level of technology (dummy 1 = general hospital; dummy 0 = teaching hospital). To examine whether large hospitals differ from small hospitals we include a variable “size of hospital” which is measured by the revenues of each hospital. We also control for the percentage of hospital revenues that is part of the competitive segment because we expect that hospitals that have a larger proportion of revenues in the competitive segment expand the B-segment more easily. Finally, to control for case mix differences, we include “the percentage of patients older than 65” as a proxy variable.

Table 2 presents the descriptive statistics of the model variables for 2006 and 2008. The model uses 8723 observations from 110 DBCs produced by 72 hospitals in 2 years. This database consists of 40 general and 32 teaching hospitals. The average number of DBCs slightly increased from 89 in 2006 to 95 in 2008. The volume on DBC level varies per hospital. The average cost per DBC was €1040 in 2006 and €1016 in 2008. The average total DBC cost decreased from €75,879 in 2006 to €74,793 in 2008. The revenues of Dutch hospitals ranged from €28 billion for the smallest hospital to more than €357 billion for the largest hospital. The average hospital market HHI is 0.276 (SD is 0.243). In general, markets with a concentration index more than 0.18 are concentrated markets [33]. This means that the Dutch hospital market is concentrated. However, hospital concentration is differentiated and varies between 0.038 and 1. The average hospital-insurer concentration index is 0.383 and varies between 0.177 and 0.641, showing that hospital-insurer concentration is even stronger than hospital concentration. This could be a result of the former leading region representative positions of health insurers [35].

**Table 2:** Descriptive statistics

Variable	*n*	Mean	Std. Dev	Min	Max
Year 2006					
DBC volume DBC volume (ln) Average cost Average cost (ln) Average # activities Average # activities (ln) DBC total costs DBC total costs (ln) Dummy cardiology Dummy dermatology Dummy gastroenterology Herfindahl index hospitals Health insurer concentration Percentage B-segment Total revenues (ln) Dummy type of hospital Age > 65 years	4373 4373 4373 4373 4373 4373 4373 4373 4373 4373 4373 4373 4373 4373 4373 4373 4373	89.36 3.25 €1040.48 5.94 18.77 2.27 €75,879.00 9.22 0.48 0.13 0.09 0.277 0.384 0.087 18.50 0.664 0.146	155.25 1.75 €1508.61 1.48 24.61 1.17 €228,970.20 2.18 0.50 0.35 0.29 0.244 0.100 0.022 0.57 0.472 0.019	1 0 €31.57 3.45 1 0 €31.57 3.45 0 0 0 0.038 0.177 0.043 17.16 0 0.067	2076 7.64 €11,032.46 9.31 223 5.40 €4,774,042.00 15.38 1 1 1 1 0.641 0.177 19.56 1 0.208
Year 2008					
DBC volume DBC volume (ln) Average cost Average cost (ln) Average # activities Average # activities (ln) DBC total costs DBC total costs (ln) Dummy cardiology Dummy dermatology Dummy gastroenterology Herfindahl index hospitals Health insurer concentration Percentage B-segment Total revenues (ln) Dummy type of hospital Age > 65 years	4350 4350 4350 4350 4350 4350 4350 4350 4350 4350 4350 4350 3791 4285 4289 4350 4350	95.04 3.33 €1,016.64 6.00 19.69 2.39 €74,793.56 9.30 0.49 0.13 0.09 0.277 0.384 0.087 18.56 0.670 0.156	159.87 1.77 €1457.10 1.41 24.19 1.13 €210,597.00 2.13 0.50 0.34 0.29 0.244 0.100 0.022 0.97 0.470 0.064	1 0 €39.84 3.68 1 0 €40.87 3.71 0 0 0 0.038 0.177 0.043 12.08 0 0.107	1856 7.53 €10,908.58 9.30 234.37 5.46 €2,689,483.00 14.80 1 1 1 1 0.641 0.177 19.69 1 0.687

Table 3 shows the changes in volume, activities and average costs between 2006 and 2008 of the treatment and control group DBCs. The care volume of two-thirds of the DBCs in both groups has increased between 2006 and 2008. The average cost decreased in both segments. In the B2 segment, for seven out of the 11 DBCs, average costs decreased. An exception is ulcerative colitis, with a cost increase of more than 15 %. For eight of the ten DBCs in the B3 segment, average costs decreased. Activity changes differ between the segments and within the specialties, indicating that performing fewer activities does not automatically lead to a cost decrease. For example, activities increased for carpal tunnel syndrome, other cardiac arrhythmia originating in the cardiac atrium and cardiac atrium fibrillation, but total costs of these DBCs decreased. This indicates a shift from more expensive activities to less expensive activities and possibly a substitution or a shift from inpatient treatments to outpatient or daycare treatments.

**Table 3:** Change in volume, average cost, activities, and total cost in 2006–2008 for treatment group (B2) and control group (B3) DBCs

Segment	Specialty	Diagnosis	Volume change 2006–2008 (%)	Activity change 2006–2008 (%)	Total cost change 2006–2008 (%)	Average cost change 2006–2008 (%)
B2	Orthopedics	Carpal tunnel syndrome	−0.56	14.01	−8.77	−3.76
B2	Orthopedics	Meniscal injury	−0.40	2.86	1.94	2.59
B2	Orthopedics	Anterior cruciate ligament injury	−8.41	−5.32	−7.60	7.58
B2	Dermatology	Psoriasiform dermatoses	19.87	−4.21	1.01	−18.31
B2	Gastroenterology	Inflammatory bowel disease	20.40	−3.44	−0.21	−5.80
B2	Gastroenterology	Ulcerative colitis	20.92	9.63	3.52	15.99
B2	Cardiology	Chronic heart failure	88.20	7.50	71.35	4.91
B2	Cardiology	Cardiac atrium fibrillation	61.01	6.20	68.74	−2.00
B2	Cardiology	Other cardiac arrhythmia originated in cardiac atrium	60.91	−28.06	49.30	−30.68
B2	Cardiology	Cardiac impulse disorder	43.86	−1.65	25.10	−8.32
B2	Cardiology	Other cardiac arrhythmia	−16.03	4.83	−21.74	−8.80
B3	Orthopedics	Cervical stenosis with myelopathy	42.67	18.44	63.73	3.22
B3	Orthopedics	Spinal stenosis	39.46	−7.13	−0.11	−8.31
B3	Orthopedics	Loosening/infection malposition knee arthroplasty	31.19	2.75	13.44	1.84
B3	Dermatology	Malignant dermatosis	6.02	6.90	11.49	−6.29
B3	Dermatology	Ulcus cruris	−2.84	−13.44	−15.09	−0.90
B3	Cardiology	Angina pectoris (stable)	13.68	6.65	−10.35	−4.31
B3	Cardiology	Angina pectoris (unstable)	−14.17	4.22	−8.90	−5.88
B3	Cardiology	Chronic heart failure	31.10	3.06	−6.15	−6.63
B3	Cardiology	Cardiac atrium fibrillation	−5.27	7.10	−8.89	−11.10
B3	Cardiology	Other cardiac arrhythmia originated in cardiac atrium	16.03	7.54	13.76	−10.85

### Empirical model

To identify the impact of the introduction of market coordination we estimated two difference-in-difference (DiD) models. We examined the impact of market competition on four dependent variables at the hospital level: production volume, number of activities, total costs and average costs, using the following model:

$$y_{ijt} = \alpha + \beta_{1} I\,({\text{Year}} = 2008)_{t} + \beta_{2} {\text{B}}2_{i} + \beta_{3} {\text{B}}2_{i} \times I\left( {{\text{Year}} = 2008} \right)_{t} + \gamma X_{jt} + \mu_{j} + \varepsilon_{ijt} ,{\text{Model I}}$$

where $y_{ijt}$ is the dependent variable for DBC *i* in hospital *j* at time *t*. *α* is the intercept. *I* is the year dummy with value 0 for 2006 and 1 for 2008. B2 is the market competition variable, which takes the value 1 if DBC *i* is part of the B2 segment and 0 if DBC *i* is part of the B3 segment. A DBC is produced in 2008 when the treatment is delivered by the care provider, and accepted and registered by the insurance company in that year. *X*_*jt*_ are the specialty dummies at the hospital level. The specialty dummy for Orthopedics is used as the basis and is included in the intercept. *μ*_*j*_ are the hospital fixed effects and *ε*_*jt*_ is the error term. We use adjusted standard errors to control for the clustering of DBCs on the hospital level. The coefficient capturing the impact of the introduction of market competition is *β*_3_: the effect of post-reform market coordination on the dependent variables’ DBC production volume, average DBC costs and average number of activities in DBCs. To examine the heterogeneity of hospitals we also estimate a model including hospitals’ characteristics (Model II).

Of relevance for the empirical strategy is whether the common trends assumption is satisfied. After all, violation of this assumption makes it difficult to interpret *β*_3_ as the proper treatment effect (i.e., the effect of the introduction of competition). We tested for the common trend assumption by estimating model (I) on data of 2006 and 2007 alone, with year 2008 replaced by 2007. These tests indicated that for all four outcome variables the common trend assumption cannot be rejected at the 5 % level. It should be noted, however, that for the logarithm of total volume, the coefficient was significant at the 10 % level (see “Appendix” for the results of this test). It is important to note that in our test for the common trends assumption we have only three data points available, of which two are prior to the reforms: we can construct a placebo DiD of 2006 and 2007. This placebo DiD indicates that the common trend is not violated, though it has to be added that the power of the test may be low. On the other hand, possible anticipation effects may lead us to reject the common trends assumption sooner. As was argued earlier (see “DIS data 2006–2008”), hospitals may have anticipated the change of 2008 and in response to this may have already adjusted their production decisions prior to 2008.^1^

## Results

Table 4 provides the results of the DiD models for each outcome measure: volume, activities, total costs and average costs. The results for the pooled data in Model I show a decrease in total cost, production volume and average number of activities that can be attributed to the introduction of market coordination. The reduction in volume is contrary to our expectation (hypothesis 1). Total costs are significantly lower than in the reference group, which is in accordance with hypothesis 2. The lower total costs appears to be the result of the reduction in total number of DBCs produced as well as in average number of activities per DBC. The two main drivers of lower costs are therefore a lower number of DBCs produced as well as lower care intensity per DBC. The dummies indicate that significant differences in all dependent variables exist between medical specializations and that the impact of market coordination on volume and cost may be specialization-specific.

**Table 4:** Difference-in-Difference results for volume (ln), activities (ln), average cost (ln) and total cost (ln)

**Diff-in-diff results**	**MODEL I: fixed effects**				**MODEL II: hospital characteristics**			
	**Volume (ln)**	**Activities (ln)**	**Total cost (ln)**	**Average cost (ln)**	**Volume (ln)**	**Activities (ln)**	**Total cost (ln)**	**Average cost (ln)**
Treatment (B2*year = 2008)	-0.080* (-1.85)	-0.085*** (-3.58)	-0.105** (-2.21)	-0.008 (-0.40)	-0.082* (-1.78)	-0.089*** -3.43	-0.104** (-2.05)	-0.003 (-0.16)
B2-segment	-0.163*** (-3.96)	0.671*** (26.21)	1.049*** (23.03)	1.234*** (55.40)	-0.190*** (-4.26)	0.674*** (22.34)	1.023*** (20.96)	1.234*** (49.54)
Dummy Year = 2008	0.096*** (3.81)	0.138*** (4.94)	0.112*** (4.21)	0.059*** (3.98)	0.113*** (3.48)	0.153*** (5.15)	0.135*** (3.62)	0.066*** (3.88)
Dummy Dermatology	1.161*** (23.57)	0.363*** (9.80)	0.964*** (19.86)	-0.118*** (-2.78)	1.143*** (22.30)	0.384*** (9.43)	0.960*** (17.84)	-0.109** (-2.37)
Dummy Gastroenterology	-0.109 (-1.53)	1.260*** (25.26)	-0.322*** (-4.40)	-0.191*** (-8.51)	-0.091 (-1.16)	1.277*** (22.86)	-0.305*** (-3.77)	-0.194*** (-7.45)
Dummy Cardiology	1.095*** (27.07)	1.108*** (40.37)	1.384*** (31.35)	0.294*** (14.83)	0.109*** (23.23)	1.110*** (37.78)	1.376*** (27.70)	0.294*** (13.99)
Herfindahl index hospital					-0.174 (-1.20)	-0.115 (-0.69)	-0.261 (-1.43)	-0.101 (-1.26)
Health insurer concentration					-0.062 (-0.17)	0.573* (1.73)	0.231 (0.57)	0.296* (1.69)
Percentage B-segment					-1.252 (-0.80)	1.130 (1.59)	-1.362 (-0.82)	-0.085 (-0.19)
Total revenues					0.145* (1.89)	-0.005 (-0.48)	0.154* (1.79)	0.010 (0.79)
Type of hospital					-0.368*** (-4.01)	-0.683 (-1.54)	-0.429*** (-4.37)	-0.064** (-2.37)
Age > 65 years					-2.006 (-1.06)	0.497 (0.40)	-2.174 (-0.85)	0.092** (0.07)
Constant	1.881 (62.48)	1.298 (58.00)	7.094 (204.29)	5.157 (273.24)	0.684 (0.44)	1.093 (3.05)	5.933 (3.37)	5.148 (14.53)
R-squared	0.151	0.288	0.147	0.170	0.131	0.270	0.129	0.164

Std. Err. adjusted for 72 clusters on hospital level

Model I: *y*_*ijt*_ *=* *α* *+*  *β*_*1*_ *I(Year* *=* *2008)*_*t*_ *+* *β*_*2*_ *B2*_*i*_ *+* *β*_*3*_ *B2*_*i*_ * I(Year *=* *2008)*_*t*_ *+* *γX*_*jt*_ *+* *μ*_*j*_ *+* *ε*_*ijt*_ Number of observations: 8723 Model II: *y*_*ijt*_ *=* *α* *+*  *β*_*1*_ *I(Year* *=* *2008)*_*t*_ *+* *β*_*2*_ *B2*_*i*_ *+* *β*_*3*_ *B2*_*i*_ * I(Year *=* *2008)*_*t*_ *+* *γX*_*jt*_ *+* δ*Z*_*jt*_ *+* *ε*_*ijt*_ Number of observations: 7423

* significance at the 10% level ** significance at the 5% level *** significance at the 1% level t-value in parentheses

The results of Model II indicate that the treatment effects comparing to our basic regression in Model I are virtually identical. Amongst the observed hospital characteristics, type of hospital is the most important. The relation between type of hospital and the dependent variables are negative, which means that general hospitals have a lower average volume, lower average number of activities, and lower average and total costs compared with teaching hospitals. The level of technology differs among the types of hospitals.

The introduction of market coordination may work out differently for different types of health care services. We therefore partitioned our data into three groups: outpatient care, daycare, and inpatient care. The results in Table 5 show that the introduction of market coordination has led to increased efficiency in outpatient treatments (see Panel A), where a reduction in number of activities also led to lower average cost per DBC. The number of daycare DBCs is lower in the experimental group, without any significant effect on total and average costs (Panel B). Average cost of inpatient DBCs is significantly higher for the market competition group, while there is no significant difference in volume or activities (refer to Panel C). This indicates that only patients with more complicated, and hence more expensive, conditions are hospitalized; relatively more patients with fewer complications and similar diseases may be treated in daycare and in policlinics.

**Table 5:** Different health care types

	**Volume (ln)**	**Activities (ln)**	**Total cost (ln)**	**Average cost (ln)**
**Panel A: Outpatient DBCs**				
Treatment (B2*year = 2008)	0.008 (0.14)	-0.070** (-2.25)	-0.074 (-1.17)	-0.078*** (-3.61)
B2-segment	0.136** (2.48)	-0.081*** (-3.51)	0.111* (1.89)	0.059*** (2.95)
Dummy Year = 2008	0.032 (0.70)	0.218*** (7.00)	0.157*** (3.65)	0.290*** (16.78)
Dummy Dermatology	2.593*** (47.33)	0.650*** (20.71)	2.584*** (41.42)	0.094*** (3.45)
Dummy Gastroenterology	0.065 (0.75)	1.566*** (24.40)	0.140 (1.40)	0.013 (0.36)
Dummy Cardiology	1.867*** (29.82)	0.863*** (28.11)	1.809*** (25.13)	-0.150*** (-5.73)
Constant	1.558 (32.17)	1.371 (62.85)	6.755 (131.24)	5.099 (276.02)
R-squared	0.439	0.583	0.423	0.264
**Panel B: Daycare DBCs**				
Treatment (B2*year = 2008)	-0.198* (-1.88)	-0.043 (-1.00)	-0.160 (-1.51)	0.040 (1.32)
B2-segment	-0.222* (-0.184)	-0.192*** (-4.81)	-0.246* (-1.92)	-0.030 (-0.93)
Dummy Year = 2008	0.240*** (3.05)	0.039 (0.80)	0.234*** (2.90)	-0.004 (-0.12)
Dummy Dermatology	-0.266 (-1.65)	0.138** (2.01)	-0.877*** (-5.11)	-0.564*** (-8.83)
Dummy Gastroenterology	-0.301** (-2.25)	1.228*** (18.85)	-0.839*** (-6.19)	-0.444*** (-11.47)
Dummy Cardiology	-0.557*** (-3.91)	0.530*** (10.39)	-1.207*** (-8.13)	-0.580*** (-15.14)
Constant	2.011 (18.20)	2.639 (70.18)	9.176 (78.58)	7.092 (261.91)
R-squared	0.072	0.431	0.116	0.369
**Panel C: Inpatient DBCs**				
Treatment (B2*year = 2008)	-0.019 (-0.33)	-0.006 (-0.16)	0.041 (0.71)	0.061* (1.95)
B2-segment	-1.227*** (-18.54)	-2.967*** (-9.74)	-1.329*** (-20.20)	-0.057* (-1.83)
Dummy Year = 2008	0.007 (0.16)	0.058 (1.28)	-0.076 (-1.59)	-0.078*** (-3.59)
Dummy Dermatology	-1.932*** (-17.78)	0.700*** (9.64)	-1.901*** (-16.36)	0.073 (1.53)
Dummy Gastroenterology	0.073 (0.74)	1.287*** (21.95)	-0.001 (-0.01)	-0.019 (-0.41)
Dummy Cardiology	1.141*** (15.69)	0.520*** (14.84)	0.556*** (7.34)	-0.528*** (-20.37)
Constant	2.641 (36.33)	3.200 (98.62)	10.693 (146.67)	7.959 (326.76)
R-squared	0.412	0.391	0.347	0.306

Std. Err. adjusted for 72 clusters on hospital level

Model I: *y*_*ijt*_ *=* *α* *+*  *β1I(Year* *=* *2008)t* *+* *β2B2*_*i*_ *+* *β3B2*_*i*_ * I(Year *=* *2008)*_*t*_ *+* *γXjt* *+* *μj* *+* *εijt*

Number of observations inpatient: 2228

Number of observations outpatient: 2401

Number of observations daycare: 1814

* significance at the 10% level ** significance at the 5% level *** significance at the 1% level t-value in parentheses

### Effects on specialty level: orthopedics and cardiology

The higher number of significant specialization dummies in Table 4 already signals that the effects of the introduction of market coordination may be different across medical specializations. For example, specializations may differ on the choice of care type, substituting inpatient care with daycare and outpatient treatments. We therefore performed analyses at the specialty level, examining whether the introduction of competition changes treatment choices (and thus costs). We selected for this analysis the medical specialties orthopedics and cardiology, because they are represented in both the experimental as well as in the control group. One advantage is that within cardiology, three diagnoses exist for which different treatment choices (DBCs) are available. For instance, DBCs of the diagnosis of chronic heart failure can be found both in the B2 segment (where competition was introduced in 2008) and the B3 segment (no competition). For orthopedics, we do not have this ‘ideal’ set-up of different treatments for a given diagnosis that are in both segments. We therefore used different diagnoses within the specialty, some of which are exposed to market competition and some which are not. For both specialties, we examined the effect of competition on the share of inpatient, daycare and outpatient treatments used for a given diagnosis. Table 6 shows the results of the regression analysis of inpatient, daycare and outpatient share and average costs (ln) for cardiology and orthopedics DBCs.

**Table 6:** Difference-in-difference results for share of outpatient care, daycare and inpatient care for cardiology and orthopedics

	**Share Outpatient**	**Share Daycare**	**Share Inpatient**	**Average cost (ln)**
**Panel A: Cardiology**				
Treatment (B2*year = 2008)	0.003 (0.31)	0.039* (1.95)	-0.042** (-2.01)	-0.200*** (-2.74)
B2-segment	-0.288*** (-42.79)	-0.007 (-0.59)	0.796*** (57.60)	2.479*** (39.76)
Dummy Year = 2008	0.009 (1.38)	0.001 (0.28)	-0.010* (-1.70)	0.115*** (5.95)
Cardiac atrium fibrillation	-0.018*** (-15.03)	0.049*** (7.86)	0.032*** (4.16)	-0.261 (-13.15)
Other cardiac arrhytmia originated in cardiac atrium	0.020*** (2.96)	0.017** (2.38)	-0.037*** (-4.37)	-0.244 (-10.81)
Constant	0.318 (63.61)	-0.026 (-5.94)	0.208 (34.37)	5.525 (283.30)
R-squared	0.198	0.134	0.802	0.509
**Panel B: Orthopedics**				
Treatment (B2*year = 2008)	0.045*** (2.73)	-0.064*** (-4.12)	0.023** (2.59)	0.106** (2.22)
B2-segment	0.309*** (20.23)	0.521*** (34.74)	0.167*** (22.25)	2.905*** (72.09)
Dummy Year = 2008	0.016* (1.79)	-0.002 (-1.59)	-0.016** (-2.38)	0.000 (0.01)
Spinal stenosis	-0.036 (-0.87)	0.005 (0.73)	-0.040 (-1.51)	0.250* (1.81)
Carpal tunnel syndrome	-0.459*** (-10.12)	0.059*** (4.80)	-0.178*** (-6.51)	-1.571*** (-10.86)
Loosening / infection malposition knee arthroplasty	-0.260*** (-6.21)	0.009*** (2.14)	0.210*** (7.18)	1.150*** (7.67)
Meniscal injury	-0.527*** (-13.24)	0.114 (16.42)	-0.166*** (-6.01)	-0.992*** (-6.78)
Anterior cruciate ligament injury	-0.441*** (-10.88)	-0.185*** (-20.63)	0.048* (1.77)	-0.957*** (-6.51)
Constant	0.531 (12.75)	0.029 (6.14)	0.070 (2.63)	4.929 (34.28)
R-squared	0.330	0.734	0.369	0.519

Std. Err. adjusted for 72 clusters on hospital level

Model I: *y*_*ijt*_ *=* *α* *+*  *β1I(Year* *=* *2008)t* *+* *β2B2*_*i*_ *+* *β3B2*_*i*_ * I(Year *=* *2008)*_*t*_ *+* *γXjt* *+* *μj* *+* *εijt*

Number of observations Cardiology: 1151 Orthopedics: 1482

Selection of diagnoses including both B2 & B3 DBCs

* significance at the 10% level ** significance at the 5% level *** significance at the 1% level t-value in parentheses

The results of the two medical specialties differ greatly. For cardiology, we see a decrease in the share of inpatient treatments (Panel A in Table 6). We also see an increase in the share of daycare treatments. It is not likely that the composition of the pool of patients changed in our observation period (2006–2008). Therefore, a possible explanation for the observed shift in shares is that for a given diagnosis, more expensive inpatient treatments are replaced by cheaper daycare treatments. As a result, the average costs of treatment for a given diagnosis declines. For orthopedics, we find different results (see Panel B). In response to the market competition introduction, the proportion of inpatient treatments and outpatient treatments increased. As a result, the average costs for the diagnosis increased. The proportion of daycare treatments decreased. These analyses show that it is difficult to predict in advance whether market competition leads to lower average costs. We find that the effect of market competition differs between specialties.

Two mechanisms may explain this finding. First, differences in price–cost margins of inpatient, and daycare and outpatient procedures at the diagnosis level may explain physician treatment choices. Unfortunately, we do not have access to price information at the diagnosis level to verify this. Second, the composition of the patient pool may have changed over time. Note that we only use data of a relatively short time span (2006–2008), so it is not likely that the patient case mix changed dramatically. Alternatively, physicians may have influenced the composition of the patient pool endogenously (by choice). For instance, they might change the admission criteria for treatment of a given diagnosis. The ability to do this may differ per specialty and at the diagnosis level. Note, however, that this must be differentially for B2 and B3 and one should see this reflected in the B2 (and B3) volumes. We do not observe clear volume effects in Table 5, which suggests that differences in price–cost margins may be more relevant.

## Conclusion

This paper assessed the effect of the introduction of market-based price competition on costs and volume in Dutch hospitals in the years 2006 and 2008. The gradual implementation of market competition for some products, and not for others, provided a unique opportunity to analyze the effects of market competition on hospital behavior. More specifically, we used 46 care products (DBCs) belonging to 11 diagnoses that were part of the budget system in 2006 and that went into the price competitive segment in 2008 (B2-DBCs). We compared these with 54 similar care products belonging to 10 diagnoses that stayed in the budget system (B3-DBCs). The database contains 814,192 observations (DBCs produced and invoiced), roughly equally divided between 2006 and 2008, and produced by 72 general hospitals.

Difference-in-difference estimates show that the implementation of market-based competition leads to relatively lower total costs, production volume and number of activities overall. The decrease in volume in the experimental group compared with the reference group is not what we expected. A possible explanation is that the exogenous demand of care is rather limited because of the relatively short research period. Amongst the observed hospital characteristics, type of hospital is the most important. General hospitals have a lower average volume, lower average number of activities, and lower average and total costs compared with teaching hospitals, as expected. Market concentration of hospitals does not have an impact and insurers do not use their monopsony power to selectively contract hospitals, which could be explained by the limited share of the market competitive segment in the research period.

To identify these results further, we estimated a DiD model on treatment level. Results show that the average costs for outpatient DBCs decreased due to a decrease in the number of activities per DBC. The introduction of market competition led to an increase of average costs of inpatient DBCs. Since both volume and number of activities have not changed significantly, we conclude that the cost increase is likely the result of more expensive activities.

A possible explanation for our finding is that hospitals may focus on efficiency improvements in outpatient care products that are relatively straightforward, using easily analyzable technologies.

The relative shares of inpatient and outpatient care differ between medical specializations. In orthopedics, daycare care is substituted with outpatient and inpatient care, leading to higher average inpatient costs. In cardiology, a reverse substitution has been detected, leading to a lower share of inpatients and thus lower average costs.

The Dutch government introduced market competition as an instrument to control health care cost and increase transparency and quality of care. Therefore different health care reforms have been introduced. During our research period the hospital reimbursement system was gradually implemented and quality information was not yet available, which means that not all necessary conditions for market competition were met. This could explain why the results of this research are relatively limited and sometimes differ from the expectations of government.

## Limitations of this study

There are several limitations of this study. The first limitation is inherent to the difference-in-difference methodology followed. In this approach, an experimental group of DBCs was selected along with a DBC control group. We have tried to select DBCs that are similar in a number of ways, e.g., belonging to the same medical specialization and having comparable technological complexities, and we performed a first test of the common trends assumption. The common trends assumption was not violated, but it has to be noted that our test may have suffered from low power, due to the limited number of pre-treatment years. All DBCs in both groups represent elective-care DBCs with relatively low levels of complexity. Our results may be different when more complex DBCs are considered. As the implementation of the market system in the Netherlands progresses, increasingly more complicated DBCs will be transferred from the budgeting system into the market system. These changes in the system will provide fresh opportunities to study the impact of the system change on cost and intensity of more complex health care products.

Our sample only included general hospitals, not academic hospitals and independent treatment centers, because of limitations in data access. This is an important omission, because we have seen that in certain conditions, e.g., a high-proportion A-segment production, inpatient costs have increased. This could also apply to academic hospitals. A similar restriction applies to independent treatment centers. We expect that the ITCs have economized significantly regarding outpatient treatments, which means that we may have underestimated the cost reduction effects for the sector as a whole.

Our analysis mainly focused on cost and intensity of care, and did not consider the effects of the introduction of market competition on quality of care. The main reason is that we could not find sufficiently complete and reliable quality information for our sample DBCs in the years 2006 and 2008. This is an important topic for future research, because cost and quality may be related to each other and are both controllable by health care providers.

## Appendix

See Table 7.

**Table 7:** Common trend results

**Common trend results**	**Coefficient β**_**3’**_
Volume (ln)	-0.074* (-1.77)
Activities (ln)	-0.014 (-0.59)
Total cost (ln)	-0.064 (-1.47)
Average cost (ln)	0.001 (0.06)

Std. Err. adjusted for 72 clusters on hospital level

Model I: *y*_*ijt*_ *=* *α* *+*  *β*_*1*_ *I(Year* *=* *2007)*_*t*_ *+* *β*_*2*_ *B2*_*i*_ *+* *β*_*3’*_ *B2*_*i*_ ** I(Year* *=* *2007)*_*t*_ *+* *γX*_*jt*_ *+* *μ*_*j*_ *+* *ε*_*ijt*_ Number of observations: 8812

* significance at the 10% level ** significance at the 5% level *** significance at the 1% level t-value in parentheses

## Footnotes

1 For this reason we decided to only use 2006 and 2008 to estimate our models.
